# CARE-DN: An Extension of the CARE Guidelines for Dry Needling Case Reports

**DOI:** 10.7759/cureus.101789

**Published:** 2026-01-18

**Authors:** Giorgos Tzigkounakis, Katerina Simati, Konstantinos Georgiadis, Vasileia Kostaridou, Paul Battersby, Sandra Calvo, Pablo Herrero, Adrian Kuzdzal, Johnson McEvoy, Zacarías Sánchez Milá, Jorge Velázquez Saornil, Michael Voight, Tianjun Wang, Manos Stefanakis

**Affiliations:** 1 Research, Health and Resilience Institute, Athens, GRC; 2 Medicine, National and Kapodistrian University of Athens, Athens, GRC; 3 Medicine, Aristotle University of Thessaloniki, Athens, GRC; 4 Executive Office, Acupuncture Association of Chartered Physiotherapists (AACP), London, GBR; 5 Physiatry and Nursing, Faculty of Health Sciences, University of Zaragoza, Zaragoza, ESP; 6 Clinical Research, Aragón Health Research Institute, Instituto de Investigación Sanitaria Aragón (IISA), Zaragoza, ESP; 7 College of Medical Sciences, Institute of Health Sciences, University of Rzeszów, Rzeszów, POL; 8 Rehabilitation and Physiotherapy Center, MEDFIT, Kraków, POL; 9 Physiotherapy, United Physiotherapy Clinic, Limerick, IRL; 10 Physiotherapy, NEUMUSK Group Research, Faculty of Health Sciences, Avila Catholic University, Castilla y León, ESP; 11 Physiotherapy, Pontifical University of Salamanca, Salamanca, ESP; 12 School of Physical Therapy, Belmont University, Nashville, USA; 13 Academy, London Academy of Chinese Acupuncture (LACA), London, GBR; 14 Physiotherapy, University of Nicosia, Nicosia, CYP

**Keywords:** care, care-dn, consensus report, delphi method, dry needling

## Abstract

Background

Dry needling (DN) is widely used in musculoskeletal and pain management, yet published DN case reports vary substantially in structure, terminology, and completeness, limiting reproducibility and interpretation. The objective of this study was to develop a consensus-based reporting guideline extension for DN case reports, as an extension of the CARE (CAse REport) guidelines.

Methods

A protocol was registered a priori on the Open Science Framework (OSF). A two-round modified electronic Delphi process was conducted with an international multidisciplinary panel of clinicians, researchers, and educators experienced in DN and acupuncture. Proposed DN-specific reporting items were generated through structured mapping of the CARE checklist and relevant literature. In Round 1, panellists rated item relevance and provided qualitative feedback. In Round 2, revised items were re-evaluated using a predefined consensus threshold of ≥80% agreement for ratings of 4 or 5 on a five-point relevance scale. An online implementation of the final checklist was subsequently developed and subjected to usability evaluation. Reporting of the Delphi study was guided by the Conducting and REporting of DElphi Studies (CREDES) guideline.

Results

In total, 13 voting panellists from seven countries participated, with 12 completing Round 2. Overall, 16 DN-specific items achieved the predefined consensus threshold and were integrated into the final CARE-DN checklist. The items address treatment specificity, anatomical targeting, procedural characteristics, needle specifications, imaging use, and clinician expertise. Usability evaluation of the online CARE-DN tool indicated favourable ratings for clarity, navigation, and checklist export, supporting feasibility for routine academic and clinical use.

Conclusion

CARE-DN provides the first consensus-based reporting guideline extension specifically for DN case reports. By supplementing the original CARE framework with DN-specific items, CARE-DN promotes transparent, accurate, and reproducible reporting. The checklist and accompanying online tool are intended to support authors, reviewers, and editors in improving the quality and interpretability of DN case literature.

## Introduction

Case reports contribute valuable clinical insights, particularly in fields where individualised assessment and manual procedures shape treatment outcomes. Dry needling (DN) is increasingly used in physiotherapy, sports medicine, pain rehabilitation, and musculoskeletal care. Despite growing research, there is no DN-specific guidance to standardise how interventions should be reported. This gap may result in variability in naming conventions, anatomical descriptors, needle specifications, procedural techniques, and documentation of responses. Such inconsistency affects clinical reproducibility and limits the integration of case findings into practice and research. A recent umbrella review further highlighted substantial heterogeneity in DN protocols across the literature and emphasised the need for greater standardisation to strengthen the clinical evidence base [1].

Reporting guidelines help promote completeness, transparency, and methodological rigour. The CARE (CAse REport) guidelines provide a widely adopted framework for clinical case reports across healthcare domains [2]. Extensions and adaptations to CARE have been developed for specific disciplines, including surgery [3], acupuncture [4], radiology [5], and COVID-19 [6], demonstrating the value of domain-focused specifications. To our knowledge, no reporting guideline has previously addressed DN, despite the intervention’s distinctive procedural, anatomical, and technical characteristics. Although DN shares the use of filiform needles with acupuncture, it is commonly delivered within an anatomically based biomedical framework and under different training and regulatory scopes, supporting the need for a dedicated reporting guideline.

CARE-DN was developed to fill this gap by identifying the minimum DN-specific information that should accompany a clinical case report. Our objective was to supplement the original CARE checklist with consensus-based DN-specific reporting items created through a rigorous Delphi process. The resulting checklist aims to support clinicians, authors, reviewers, and editors in improving the quality and reproducibility of DN case reports.

## Materials and methods

Study design and registration

The CARE-DN project followed a predefined protocol registered on the Open Science Framework (OSF) [7] prior to data collection (the protocol is available at: https://doi.org/10.17605/OSF.IO/PT3RJ). The methodological approach was based on established principles for guideline development and Delphi methodology [8,9], and aligned with best practices for reporting Delphi studies [10]. The design, conduct, and reporting of this Delphi study align with the Guidance on Conducting and REporting of DElphi Studies (CREDES) [10].

A separate protocol article was not published, but all methodological steps, including Delphi materials, item refinement, consensus decisions, and amendments, were prospectively documented on OSF, which serves as the public protocol record. The accompanying Explanation and Elaboration document, which provides item-level rationale, clarifications, and illustrative examples, is provided as supplementary material (see Appendix, Supplementary File S1). Patients and members of the public were not involved in the design, conduct, reporting, or dissemination plans of this Delphi-based guideline development study, which focused on expert consensus and did not include patient data.

Overview of development process

The development of CARE-DN followed a predefined, multi-phase process aligned with established principles for reporting guideline development and modified Delphi methodology. The process consisted of four sequential stages: (1) project initiation and panel formation, (2) initial item generation, (3) Delphi consensus and refinement, and (4) usability evaluation and implementation considerations. The online CARE-DN tool was developed only after consensus was finalised and did not influence item generation or retention.

Project initiation and panel composition

An international multidisciplinary Delphi panel was assembled using purposive sampling. In total, 13 panellists from seven countries participated, including academic and clinical staff affiliated with seven universities, and representatives from professional associations and specialist practices. Of these, 12 panellists had established expertise in DN through advanced practice, teaching, and peer-reviewed publications. One senior academic with expertise in Traditional Chinese Medicine (TCM) acupuncture was intentionally included to broaden disciplinary representation and provide a complementary perspective on needle-based interventions. One panellist held dual qualifications in physiotherapy and TCM acupuncture, contributing integrative expertise spanning both biomedical and traditional East Asian medical paradigms. In addition, one non-clinical medical information systems auditor supported tool development and preliminary usability testing and was not involved in Delphi voting. Table 1 summarises the characteristics of the CARE-DN Delphi panel and project contributors.

**Table 1:** Characteristics of the CARE-DN Delphi panel and project contributors Values are reported as n (%) where applicable. Percentages are calculated using the number of voting Delphi panellists as the denominator (n = 13). One additional non-clinical technical contributor participated in project development and usability assessment, but did not take part in Delphi voting, resulting in 14 total project members. Abbreviations: PhD, Doctor of Philosophy; PhD(c), Doctor of Philosophy candidate; DPT, Doctor of Physical Therapy; MSc, Master of Science.

Characteristic	Value
Voting Delphi panellists	13
Countries represented	7
Highest academic qualification (all project contributors, n = 14)	PhD, 8 (57%)
	PhD(c), 2 (14%)
	DPT, 1 (7%)
	MSc, 3 (21%)
Academic appointment (university-based)	8 (62%)
Clinical practice background	13 (100%)
≥10 years of experience with dry needling	9 (69%)
5-9 years of experience with dry needling	3 (23%)
Does not practice dry needling, senior Traditional Chinese Medicine (TCM) acupuncture expert	1 (8%)
Primary discipline: Physiotherapy	11
Primary discipline: Medicine	1
Primary discipline: Acupuncture/TCM	1
Non-clinical technical contributor (non-voting)	1

Initial item generation

The initial pool of DN-specific items was generated by the core research team prior to the Delphi process through structured mapping of the CARE checklist against published DN case reports and relevant clinical and methodological literature. Existing reporting guidance for needling and complex non-pharmacological interventions, including the CARE-Acupuncture extension and TIDieR (Template for Intervention Description and Replication) principles, also informed item generation, with emphasis on domains not adequately covered by CARE. The development group reviewed the preliminary list to remove redundancy and improve clarity. No voting occurred at this stage. The resulting item set was submitted unchanged to Round 1 for independent panel evaluation.

Delphi consensus process

A two-round modified electronic Delphi process was implemented using anonymised online surveys. In Round 1, panellists rated each proposed DN-specific checklist item on a five-point scale of relevance and provided free-text comments. Responses were analysed quantitatively and qualitatively. Free-text comments were examined using a simple descriptive content approach, in which similar remarks were grouped into recurring themes and used to refine item wording, remove redundancies, and clarify examples for the next round. Consistent with the preregistered protocol, items with at least 75% of ratings at 4 or 5 were considered to have met the preliminary consensus threshold. These items were refined on the basis of comments and carried forward to Round 2, whereas the single item that did not reach this threshold was excluded. Feedback-informed wording adjustments, removal of redundancies, and greater use of direct, action-oriented phrasing were applied.

**Figure 1:**
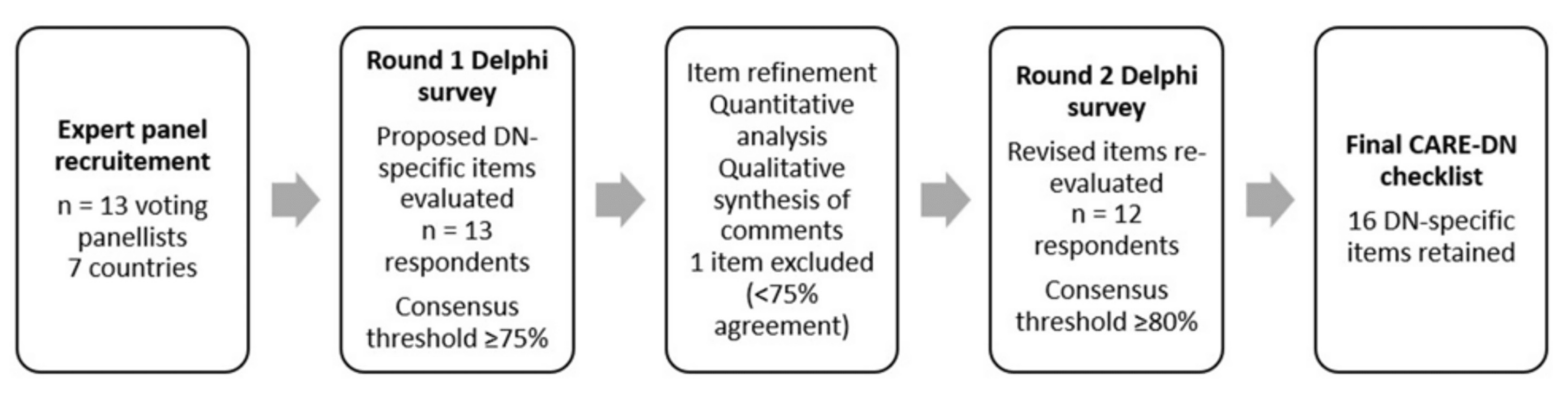
Flow diagram of the CARE-DN modified Delphi process An international expert panel participated in a two-round electronic Delphi survey to evaluate proposed dry needling-specific reporting items for inclusion in the CARE-DN checklist. In Round 1, items meeting a predefined preliminary consensus threshold of ≥75% agreement (ratings 4-5) were retained and refined based on quantitative ratings and qualitative feedback, while one item was excluded. In Round 2, revised items were re-evaluated using a predefined consensus threshold of ≥80% agreement (ratings 4-5). All 16 items meeting this threshold were retained in the final CARE-DN checklist.

In Round 2, the revised items were re-evaluated using the same five-point relevance scale. Consensus for final inclusion was defined a priori as at least 80% of panellists rating an item 4 or 5. Descriptive statistics were calculated for each item, including means, standard deviations, and interquartile ranges. All 16 items reached the predefined Round 2 threshold and were therefore retained in the final CARE-DN checklist. Figure 1 summarises the process.

Usability evaluation 

After the Delphi process, the usability of the online CARE-DN tool was assessed. Prior to structured testing, one medical doctor, one senior physiotherapist, and one IT specialist informally tested the tool and provided feedback on clarity, navigation, and workflow. Delphi panellists then used the online version of the tool for 10 days and completed a brief online questionnaire with five-point Likert items and optional free-text comments on usability, item clarity, navigation, and PDF export. The usability evaluation focused on online implementation and did not lead to changes in checklist content.

Ethical considerations

Participation in the CARE-DN Delphi was voluntary. All panellists provided electronic informed consent via an online enrolment and authorship form before the commencement of the study. No patients were involved, and no identifiable personal data were collected. Survey responses were analysed in aggregate and without individual attribution. Given the expert-only nature of the study and the absence of patient involvement, formal research ethics committee approval was not required.

## Results

Consensus outcomes

In total, 12 of 13 panellists completed Round 2. All 16 DN-specific items achieved at least 80% agreement at ratings of 4 or 5. Items demonstrated strongly positive distributions, narrow interquartile ranges, and high mean relevance scores. Panel comments supported the final item set and highlighted the need for clarity, standardisation, and practical usability.

Informal preliminary testing of the online tool by a medical doctor, a senior physiotherapist, and an IT specialist in medical systems auditing suggested that both the checklist items and the online interface were clear in practice. In a separate structured usability evaluation focused on the online implementation, Delphi panellists completed a brief questionnaire after using the tool. Ratings addressed navigation, layout, and PDF export rather than the checklist content, and were consistently favourable, with respondents describing the tool as clear, concise, and quick to use, which supports its feasibility for routine academic and clinical use. Overall, these findings indicate that the tool operationalises CARE-DN by structuring checklist completion and generating an exportable checklist file suitable for journal submission.

Final CARE-DN items

The final CARE-DN checklist supplements the original CARE guideline by adding 16 DN-specific reporting items across the domains of Title, Keywords, Abstract, Timeline, Diagnostic Assessment, and Therapeutic Interventions. These items cover essential aspects of anatomical targeting, procedural description, needle characteristics, clinician expertise, and optional imaging guidance. Together, they strengthen the specificity and reproducibility of DN descriptions in clinical case reporting. Table 2 summarises the domains and scope of the DN-specific reporting elements added to the original CARE framework. Table 3 presents the full CARE-DN checklist in an integrated format, combining the original CARE items with the DN-specific extension items. An accompanying Explanation and Elaboration document is available as Supplementary File S1.

**Table 2:** Domains and scope of the CARE-DN checklist additions Summary of the domains and scope of the dry needling-specific reporting elements added to the original CARE framework.

CARE domain	Scope of CARE-DN additions
Title	Explicit identification of the intervention as dry needling to improve specificity and indexing
Keywords	Inclusion of “dry needling” among keywords to enhance discoverability
Abstract	Clear specification of the dry needling intervention and, where relevant, the primary target or technique
Timeline	Reporting of the number of dry needling sessions delivered and the interval between sessions
Diagnostic assessment	Clinical rationale for selecting dry needling and reporting of imaging or objective assessments when used
Therapeutic intervention	Detailed description of targeted muscles or tissues, anatomical landmarks, needle specifications, number of needles, needling technique, depth and angle when relevant, imaging guidance if used, and duration of needle retention
Practitioner factors	Reporting of clinician profession, dry needling training or certification, and experience relevant to the intervention

**Table 3:** Integrated CARE and CARE-DN checklist for dry needling case reports Items 1b, 2b, 3e, 7b, 8e, 8g, 9d, 9e, 9f, 9g, 9h, 9i, 9j, 9k, 9l, and 9m, which are in bold, represent CARE-DN dry needling-specific extension items. All other items correspond to the original CARE guideline. Detailed guidance is available in the accompanying Explanation and Elaboration document (Supplementary File S1). Authors should indicate page or section numbers when submitting the checklist to journals that require location mapping.

Topic	Item	Checklist item description
Title	1a	The diagnosis or intervention of primary focus followed by the words “case report.”
	1b	Include “dry needling” in the title
Keywords	2	2 to 5 key words that identify diagnoses or interventions in this case report, including "case report"
	2b	Include “dry needling”
Abstract (no references)	3a	Introduction: What is unique about this case and what does it add to the scientific literature?
	3b	Main symptoms and/or important clinical findings
	3c	The main diagnoses, therapeutic interventions, and outcomes
	3d	Conclusion - What is the main “take-away” lesson(s) from this case?
	3e	Specify DN intervention clearly
Introduction	4	One or two paragraphs summarising why this case is unique (may include references)
Patient Information	5a	De-identified patient specific information
	5b	Primary concerns and symptoms of the patient
	5c	Medical, family, and psycho-social history including relevant genetic information
	5d	Relevant past interventions with outcomes
Clinical Findings	6	Describe significant physical examination (PE) and important clinical findings
Timeline	7a	Historical and current information from this episode of care organised as a timeline
	7b	Report the number of DN sessions delivered and the interval between sessions. If intervals changed during care, report what changed and why
Diagnostic Assessment	8a	Diagnostic testing (such as PE, laboratory testing, imaging, surveys)
	8b	Diagnostic challenges (such as access to testing, financial, or cultural)
	8c	Diagnosis (including other diagnoses considered)
	8d	Prognosis (such as staging in oncology) where applicable
	8e	Rationale for DN: clinical reasoning behind selecting DN over other treatments
	8g	Report any imaging or objective assessments used (if any) to guide diagnosis or dry needling (e.g. ultrasound, EMG, dynamometry)
Therapeutic Intervention	9a	Types of therapeutic intervention (such as pharmacologic, surgical, preventive, self-care)
	9b	Administration of therapeutic intervention (such as dosage, strength, duration)
	9c	Changes in therapeutic intervention (with rationale)
	9d	Report targeted muscles/tissues (name each muscle treated)
	9e	Report anatomical landmarks used for localisation
	9f	Report needle specifications (brand, material, gauge, length)
	9g	Number of needles used
	9h	Report depth and angle when relevant and include patient position and the approach path. Numeric depths are not required.
	9i	Report the needling technique employed (e.g., static retention, pistoning, rotation) and the duration of active manipulation and, if recorded, number of bouts or frequency
	9j	Report imaging guidance if used and include the modality, brand, model, and key settings
	9k	Report whether a local twitch response was elicited (yes/no) and, if recorded, the approximate number and location
	9l	Duration of needle retention
	9m	Clinician credentials/experience
Follow-Up and Outcomes	10a	Clinician and patient-assessed outcomes (if available)
	10b	Important follow-up diagnostic and other test results
	10c	Intervention adherence and tolerability (How was this assessed?)
	10d	Adverse and unanticipated events
Discussion	11a	A scientific discussion of the strengths AND limitations associated with this case report
	11b	Discussion of the relevant medical literature with references
	11c	The scientific rationale for any conclusions (including assessment of possible causes)
	11d	The primary “take-away” lessons of this case report (without references) in a one-paragraph conclusion
Patient Perspective	12	The patient should share their perspective in one to two paragraphs on the treatment(s) they received
Informed Consent	13	Did the patient give informed consent? Please provide if requested

Summary of panel feedback

Panellists emphasised clear definitions, objective measures, and explicit reporting of adverse events. Adverse events remain covered under CARE item 10d, and no DN-specific adverse event items were added. One panellist proposed hygiene and sterile-field practices. These were not included as checklist items because CARE-DN is a reporting guideline, not a practice standard, so such details are treated as optional contextual information in the Explanation and Elaboration document. Suggestions to improve clarity through action-oriented wording were incorporated. Recommendations related to exemplars, dissemination, and implementation were noted for post-publication work. All quantitative ratings and qualitative comments from both Delphi rounds were archived on OSF.

## Discussion

The CARE-DN extension represents the first consensus-based reporting guideline tailored specifically to DN case reports. Existing DN literature demonstrates wide variation in how key procedural elements are described, which limits reproducibility, comparability, and synthesis across studies. The final DN-specific items address domains repeatedly identified as clinically influential in the DN literature, including targeted anatomical structures, needling parameters, and manipulation techniques, as well as the clinician’s professional background and DN-specific training [11-13]. In this way, CARE-DN responds to an established gap by offering a structured, transparent, and clinically relevant framework for reporting DN interventions.

By building on the existing CARE framework, CARE-DN enhances reporting specificity for DN without modifying the foundational, well-accepted structure of CARE. As with other extensions, such as CARE-Acupuncture, the goal is to complement rather than replace the parent guideline. The emphasis on clarity, reproducibility, and procedural transparency aligns with recent recommendations for strengthening case report quality and supports more consistent clinical interpretation and future evidence synthesis [2].

Methodological rigour was strengthened through prospective registration, predefined consensus thresholds, and transparent reporting of participation. We obtained 12 of 13 responses (92%) in Round 2. Methodological guidance for Delphi surveys has long suggested ~70% per-round response as adequate for rigour [9], while published health-Delphi applications have regarded 61%-70% response rates as acceptable [14,15]. Current guidance emphasises the importance of transparently reporting participation and attrition to enable appraisal of methodological quality [10]. In this context, our 92% retention indicates excellent panel stability and engagement [10]. 

Consensus at the final round was defined a priori as ≥80% agreement (ratings 4-5). This approach follows the ACCORD (ACcurate COnsensus Reporting Document) guideline, which recommends pre-specifying and reporting consensus criteria [16]. In ACCORD’s Delphi process, consensus was ≥80% agreement among at least 20 respondents; although our panel was smaller, we adopted the same threshold and report participation and retention in full. This threshold is also consistent with recent field-specific precedent in the CARE-Acupuncture extension, which retained items at ≥80% agreement [4].

The international composition of the panel and the inclusion of clinicians, educators and researchers with substantial DN experience contribute to the external validity of the final checklist. At the same time, panel recommendations that extended beyond reporting, such as developing exemplars or providing implementation and dissemination tools, were recognised as important but were intentionally reserved for post-publication development. These components will be addressed in subsequent dissemination stages. Preliminary tool testing included review by an IT specialist in medical systems auditing, whose combined technical and usability input strengthened confidence in both the platform’s functionality and its practical applicability.

The inclusion of a usability evaluation distinguishes CARE-DN from many existing reporting guideline extensions and supports its implementation beyond publication, particularly through integration into an online, journal-ready reporting tool.

Limitations

Limitations include the modest sample size inherent to expert-based Delphi methodology and the predominantly English-speaking expertise represented. However, this panel size and composition are consistent with expert consensus development for reporting guideline extensions, and all elements required for transparent appraisal under CREDES were explicitly reported. Although we did not conduct a full pilot in published case reports or measure inter-rater reliability, preliminary clinician testing and a structured usability evaluation with the Delphi panel provide initial evidence of online tool usability, and highlight the need for future empirical assessment. Further research should evaluate the checklist’s usability and performance in real-world submissions and across different clinical and educational settings. In parallel, we plan targeted dissemination, translations, and implementation studies to support and monitor the adoption of CARE-DN in published DN case reports. Nonetheless, CARE-DN already provides a robust, consensus-based reporting structure that supports more transparent communication of DN case findings and strengthens the methodological foundation for future work in the field. As an initial implementation step, we have developed and tested a freely accessible online tool that hosts the CARE-DN and related CARE checklists and generates journal-ready PDF checklists, which may facilitate uptake by authors and editors.

## Conclusions

CARE-DN provides a consensus-based extension of the CARE guidelines for accurate, transparent, and reproducible reporting of DN case reports. The checklist was developed through a structured Delphi process and reflects international expert agreement on essential DN-specific reporting elements. CARE-DN is intended to support authors, clinicians, educators, and journal editors in enhancing the clarity and interpretability of DN case literature. The checklist and Explanation and Elaboration document will be disseminated through professional networks and submitted for registration with the EQUATOR Network to promote widespread adoption.

## Appendices

Supplementary File S1: CARE-DN - Explanation & Elaboration (E&E)

Purpose of This Document

This Explanation & Elaboration (E&E) document accompanies the CARE-DN checklist. It provides item-by-item guidance and illustrative examples to help authors apply each dry-needling (DN)-specific item consistently when reporting case reports. It should be read alongside:

- the original CARE checklist, and - the CARE-DN extension, which adds 16 DN-specific items.

The E&E explains why each DN-specific item matters, what should be reported, and offers short examples or wording templates. Examples are illustrative, not prescriptive. Authors should adapt them to their own clinical context and journal requirements. An online implementation of the CARE-DN checklist is available to support structured checklist completion and generation of a journal-ready reporting file (access details: care.healthandresilience.org)

“Dry needling” as an umbrella term

Always use dry needling as the core term. This ensures clarity, indexing and consistency across reports. Where helpful, a single modifier may be added (for example, trigger point dry needling or intramuscular dry needling) to clarify the primary anatomical target or technique, provided the terminology is used consistently in the title, abstract, keywords and methods.

Hygiene and anatomical considerations (contextual reporting)

CARE-DN is a reporting guideline, not a practice standard. It does not prescribe how DN should be performed or which infection-control procedures must be used. However, authors may report contextual factors that influence safety or interpretation, for example:

- skin preparation or sterile/clean field use, - special precautions in deep or anatomically complex regions, - factors relevant to adverse events.

Such details are optional and should be included when they clarify the clinical context, particularly in relation to item 10d of the original CARE guideline (adverse and unanticipated events), not as separate checklist items.

Title

Item 1b - Include “dry needling” in the title

Explanation: Including dry needling in the title makes the intervention explicit, improves indexing and helps readers and reviewers recognise DN case reports quickly. This directly supports discoverability and linkage with DN literature and MeSH indexing.

Example: “Resolution of chronic plantar heel pain after dry needling: A case report”

If a single modifier is central to the case, authors may optionally use expressions such as trigger point dry needling or perineural dry needling.

Keywords

Item 2b - Include “dry needling” in the keywords

Explanation: Standardised keywords enhance searchability and facilitate identification of DN-related case reports. Including dry needling as a keyword aligns with indexing conventions and helps systematic reviewers and database searches. Dry Needling is also an official MeSH descriptor (UI: D000079245), so its inclusion supports accurate indexing in biomedical databases.

Example: Keywords: dry needling, myofascial pain, shoulder, case report

Abstract

Item 3e - Specify the DN intervention clearly in the abstract

Explanation: The abstract is often the only part many readers see. Explicitly naming DN and briefly specifying the type of DN intervention improves clarity and discoverability. Therefore, within tight word limits, DN should be named clearly and, when central to interpretation, a concise target or technique modifier may be used.

Practical guidance:

Always name dry needling in the abstract.

Where essential, add one short modifier (for example intramuscular dry needling, intratendinous dry needling, ultrasound-guided dry needling), ensuring terminology matches the detailed report in items 9d-9m.

Example: “Treatment consisted of ultrasound-guided intratendinous dry needling and exercise therapy.”

Timeline

Item 7b - Report the number of DN sessions delivered and the interval between sessions. If intervals changed during care, report what changed and why.

Explanation: Reporting how many DN sessions were delivered and how frequently they occurred provides essential context for treatment dosage and allows readers to compare cases and outcomes. Changes in planned frequency can affect interpretation (for example, early improvement leading to fewer sessions, or interruptions due to illness or travel). This information also enhances reproducibility, enabling other clinicians and researchers to approximate the treatment dose and schedule used in the case.

What to report: - Total number of DN sessions. - Planned interval (for example weekly, twice monthly). - Actual intervals if they changed, plus a brief explanation if this is relevant to interpretation. - Ensure consistency with the timeline in CARE item 7a.

Example: “Six DN sessions were planned at weekly intervals. The patient attended four sessions over 6 weeks due to an intercurrent respiratory infection and work-related travel.”

Diagnostic Assessment

Item 8e - Rationale for DN: clinical reasoning behind selecting DN over other treatments

Explanation: Describing why DN was chosen over alternative options improves transparency, supports evidence-based reasoning and helps readers understand the context in which DN was used.

What to report: - Why DN was considered appropriate at this stage. - Relevant prior interventions and their results (for example limited response to manual therapy or exercise) - if applicable. - Any key mechanistic rationale (for example targeting a palpable trigger point or tendinopathic lesion). - Patient-specific factors influencing the choice (for example comorbidities, contraindications, preferences).

Example template: “We selected dry needling to address [target or impairment] after [relevant prior care or context] because [reason DN was suitable for this case].”

Example: “We selected dry needling to address proximal patellar tendinopathy after limited response to progressive loading, as ultrasound-guided intratendinous needling targeted the degenerative focus and the patient declined injection therapy.”

Item 8g - Report any imaging or objective assessments used (if any) to guide diagnosis or dry needling (e.g. ultrasound, EMG, dynamometry).

Explanation: This item applies only when such tools were used. Its purpose is to make transparent any imaging or objective assessments that informed diagnosis or the decision to use DN, or that were used to monitor response. It does not require the use of imaging or devices.

What to report: - Type of tool (for example B-mode ultrasound, EMG, dynamometer, pressure algometer). - Purpose (for example confirming diagnosis, identifying target tissue, monitoring strength or pain thresholds) - For imaging, a brief indication of key technical aspects relevant to interpretation, such as transducer type and frequency, depth range or mode, recognising that detailed device reporting for procedural guidance is captured more specifically under item 9j.

Example: “Diagnostic ultrasound (B-mode, 12 MHz linear probe, depth 3 cm) demonstrated focal hypoechoic thickening of the proximal patellar tendon, which guided the decision to perform intratendinous DN.”

Therapeutic intervention

Item 9d - Report targeted muscles/tissues (name each muscle treated)

Explanation: Accurate replication requires explicit identification of all muscles or other tissues treated. Broad descriptions such as “shoulder muscles” are insufficient. Anatomical clarity also helps contextualise outcomes and any adverse events.

What to report: - Each muscle or structure treated by name. - Non-muscular targets (for example tendon, scar tissue, peri-neural tissues) where applicable.

Example: “Dry needling was applied to the upper trapezius, infraspinatus and supraspinatus muscles.”

Item 9e - Report anatomical landmarks used for localisation

Explanation: Anatomical landmarks help readers understand how the target was located, particularly in deep or complex regions. This supports reproducibility and allows appraisal of anatomical safety.

What to report: - Key surface or palpable landmarks used to localise the target. - When relevant, any notable anatomical variations encountered.

Example: “The trigger point in the upper trapezius was localised midway between the C7 spinous process and the lateral third of the clavicle, with the patient seated.”

Item 9f - Report needle specifications (brand, material, gauge, length)

Explanation: Needle characteristics influence depth, manipulation and patient comfort. Reporting brand and physical characteristics supports replication and interpretation of safety considerations.

Example: “Single-use stainless steel needles (Seirin J-Type, 0.25 × 40 mm) were used for all DN applications.”

Item 9g - Number of needles used

Explanation: The number of needles used in a session is part of the overall treatment dose and may influence both clinical effect and risk of adverse events. Reporting it contributes to a clearer picture of the intervention, especially when multiple muscles or regions are treated.

Example: “Three needles were inserted in the upper trapezius and two in the infraspinatus during each session (total of five needles per session).”

Item 9h - Report depth and angle when relevant and include patient position and the approach path. Numeric depths are not required.

Explanation: Depth, angle and approach path are important for understanding both therapeutic intent and anatomical risk, particularly near neurovascular structures, pleura or viscera. Numeric depth values are not mandatory, but enough detail should be provided to aid interpretation and adverse event assessment.

What to report: - Patient position (for example prone, supine, side-lying, seated). - General depth (for example “superficial to fascia”, “to muscle belly”, “to bone contact”, “approximately one-third of needle length”). - Direction or approach path (for example medial-to-lateral, caudal-to-cranial).

Example: “With the patient prone, the needle was inserted perpendicular to the skin into the upper trapezius to the depth of the muscle belly, directed slightly caudally, with care to avoid the lung field.”

Item 9i - Report the needling technique employed (e.g., static retention, pistoning, rotation) and the duration of active manipulation and, if recorded, number of bouts or frequency.

Explanation: Different DN techniques (for example fast-in/fast-out pistoning, static retention, rotational techniques) and the duration/frequency of active manipulation may influence outcomes and adverse effects. Authors are asked to report both the technique and the duration of manipulation and, when recorded, the number of bouts or frequency.

What to report: - Technique (for example static retention, fast-in/fast-out pistoning, rotation). - Duration of active manipulation (for example seconds of pistoning). - If recorded, number of bouts or repetitions.

Example: “A fast-in/fast-out pistoning technique was used for approximately 20 seconds per point, with two to three bouts per needle, followed by static retention for 2 minutes.”

Item 9j - Report imaging guidance if used and include the modality, brand, model, and key settings.

Explanation: This item applies only when imaging is used to guide the procedural delivery of DN (for example ultrasound-guided peritendinous or intratendinous needling). Transparent reporting of imaging guidance supports reproducibility and safety appraisal. Brand, model and key settings shall be reported.

What to report: - Imaging modality (for example ultrasound, fluoroscopy). - Device brand and model.

Key settings that materially affect procedure (for example, probe type and frequency, depth, main mode or specific safety-related feature).

Example: “Patellar tendon dry needling was performed under real-time ultrasound guidance (GE Logiq e, 12 MHz linear transducer, depth 3 cm, B-mode, focus at the proximal tendon). The needle path and tip were visualised throughout the procedure.”

This example shows clearly that authors should specify modality, brand, model and key settings.

Item 9k - Report whether a local twitch response was elicited (yes/no) and, if recorded, the approximate number and location.

Explanation: The presence or absence of a local twitch response (LTR) is widely reported in DN practice and may be clinically relevant in some protocols. Reporting whether an LTR occurred, and where, helps readers understand how the technique was applied and may enable future analyses on the relationship between LTR and outcomes.

What to report: - Whether an LTR occurred (yes/no). - If recorded, approximate number and muscles/regions in which LTRs occurred.

Example: “Two local twitch responses were elicited in the infraspinatus and one in the upper trapezius during the first session. No LTRs were observed in subsequent sessions.”

Item 9l - Duration of needle retention

Explanation: In protocols using static needle retention, the length of time the needle is left in situ may influence effects and patient tolerance. Reporting retention time contributes to understanding treatment dose.

What to report: - Approximate retention time per needle or per region. - Whether retention time varied across sessions (if relevant to interpretation).

Example: “After active manipulation, needles were retained in situ for approximately 5 minutes in each treated muscle.”

Item 9m - Clinician credentials/experience

Checklist text “Clinician credentials/experience.”

Explanation: Clinician professional background, DN training and experience may influence both outcomes and safety, particularly for complex anatomical regions or imaging-guided procedures. This item does not serve as a credentialing standard but promotes transparency about who delivered the intervention.

What to report: - Professional role (for example physician, physiotherapist). - DN-specific training or certification (for example accredited course, postgraduate module). - Approximate years of DN practice or experience.

Example: “The intervention was delivered by a physiotherapist with 10 years of musculoskeletal practice and 7 years of DN experience, including completion of an accredited 50-hour DN training programme.”

Closing note

The CARE-DN E&E is intended to be used alongside the CARE-DN checklist and the original CARE guideline. Together, they support more transparent, complete and reproducible reporting of dry needling case reports and provide a structured foundation for future evidence synthesis and methodological improvement in DN research.
